# Classification and Regression Tree (CART) analysis to predict influenza in primary care patients

**DOI:** 10.1186/s12879-016-1839-x

**Published:** 2016-09-22

**Authors:** Richard K. Zimmerman, G. K. Balasubramani, Mary Patricia Nowalk, Heather Eng, Leonard Urbanski, Michael L. Jackson, Lisa A. Jackson, Huong Q. McLean, Edward A. Belongia, Arnold S. Monto, Ryan E. Malosh, Manjusha Gaglani, Lydia Clipper, Brendan Flannery, Stephen R. Wisniewski

**Affiliations:** 1 University of Pittsburgh, Pittsburgh, PA USA; 2 Marshfield Clinic Research Foundation, Marshfield, WI USA; 3 Centers for Disease Control and Prevention, Atlanta, GA USA; 4 Baylor Scott & White Health, Texas A&M Health Science Center College of Medicine, Temple, TX USA; 5 Group Health Cooperative, Seattle, WA USA; 6 University of Michigan, Ann Arbor, MI USA; 7 Department of Family Medicine, University of Pittsburgh, 3518 5th Avenue, Pittsburgh, PA USA; 8 UPMC Urgent Care - Natrona Heights, Natrona Heights, PA USA

**Keywords:** Clinical decision tools, Influenza, Recursive partitioning

## Abstract

**Background:**

The use of neuraminidase-inhibiting anti-viral medication to treat influenza is relatively infrequent. Rapid, cost-effective methods for diagnosing influenza are needed to enable appropriate prescribing. Multi-viral respiratory panels using reverse transcription polymerase chain reaction (PCR) assays to diagnose influenza are accurate but expensive and more time-consuming than low sensitivity rapid influenza tests. Influenza clinical decision algorithms are both rapid and inexpensive, but most are based on regression analyses that do not account for higher order interactions. This study used classification and regression trees (CART) modeling to estimate probabilities of influenza.

**Methods:**

Eligible enrollees ≥ 5 years old (*n* = 4,173) who presented at ambulatory centers for treatment of acute respiratory illness (≤7 days) with cough or fever in 2011–2012, provided nasal and pharyngeal swabs for PCR testing for influenza, information on demographics, symptoms, personal characteristics and self-reported influenza vaccination status.

**Results:**

Antiviral medication was prescribed for just 15 % of those with PCR-confirmed influenza. An algorithm that included fever, cough, and fatigue had sensitivity of 84 %, specificity of 48 %, positive predictive value (PPV) of 23 % and negative predictive value (NPV) of 94 % for the development sample.

**Conclusions:**

The CART algorithm has good sensitivity and high NPV, but low PPV for identifying influenza among outpatients ≥5 years. Thus, it is good at identifying a group who do not need testing or antivirals and had fair to good predictive performance for influenza. Further testing of the algorithm in other influenza seasons would help to optimize decisions for lab testing or treatment.

## Background

Influenza and other respiratory viruses cause an enormous healthcare burden in the U.S. Each year, influenza alone is responsible for 226,000 (54,000–431,000) hospitalizations [1] and 31.4 million outpatient visits [2]. Appropriate diagnosis and cost-effective treatment are dependent upon timely presentation for care, accurate and reasonably priced testing with short turnaround time, when appropriate. The U.S. Centers for Disease Control and Prevention (CDC) recommends antiviral treatment with a neuraminidase inhibitor for all outpatients with suspected or confirmed influenza who are at higher risk for influenza complications because of age or underlying medical conditions, when treatment can be started within 48 h of illness onset. Treatment also can be considered on the basis of clinical judgment, for outpatients with uncomplicated, suspected influenza who are not at increased risk for developing complicated illness, if antiviral treatment can be initiated within 48 h of illness onset.

Use of antiviral medications, specifically neuraminidase inhibitors, within 2 days of illness onset, has been shown to reduce the time to alleviation of symptoms by about half a day to a day, depending on the authority cited [3–5], and the risk of secondary complications of influenza such as clinically diagnosed pneumonia in some meta analyses [6, 7], but not in others [3, 4]. Prescribing of antiviral medications among primary care clinicians for treatment of outpatient acute respiratory infections (ARI) is infrequent [8, 9], perhaps because of cost and/or side effects associated with neuraminidase inhibitors [3, 4, 6].

Because these medications are effective only against influenza, they should only be prescribed to patients with suspected or confirmed influenza. Without respiratory viral testing, differentiating influenza from other viral respiratory infections can be difficult. Although molecular multi-viral respiratory panel testing can accurately distinguish possible causes of ARI, these tests are relatively new and expensive. Rapid influenza tests, in contrast, are inexpensive but lack sensitivity [10]. Moreover, clinical experience suggests that the use of respiratory viral testing increases as influenza circulation increases in the community, but is ordered less frequently at the beginning and end of the influenza season.

Clinicians use medical clinical decision algorithms, based on a series of decision rules, to determine risk for a range of medical conditions [11]. The availability of a clinical decision algorithm for determining influenza would benefit patients and potentially minimize costs to the healthcare system by providing the opportunity to diagnose influenza with reasonable accuracy and potentially treat patients with antiviral medication.

This study was designed to determine if an algorithm developed using recursive partitioning resulted in reasonable estimates of the likelihood of ARI being due to influenza infection. Classification and regression trees have been used to analyze mortality in persons with influenza A (H5N1) virus [12] and the likelihood of seasonal influenza [13, 14]. In one study of seasonal influenza, the presence of influenza was most accurately predicted by a model that assigned scores to symptoms; it included fever plus cough, myalgia, duration <48 h and chills or sweats [13]. Using the same data set, but grouping specific symptoms into dichotomous variables (present/absent), a model was developed in which the presence of both temperature >37.3 °C and chills or sweats predicted high risk of influenza [14]. Absence of both symptoms predicted low risk, and presence of only temperature >37.3 °C predicted moderate risk. In the present study, the Classification and Regression Trees [15] (CART) methodology was used to estimate the likelihood of influenza among individuals presenting for outpatient care for ARI in 2011–2012 who were enrolled in the U.S. Influenza Vaccine Effectiveness Network (US Flu VE Network) study.

## Methods

### Enrollment

Details of the US Flu VE Network design, sites, and enrollment procedures have been described previously [16]. Briefly, during the 2011–2012 influenza season, patients aged ≥6 months seeking outpatient medical care for an ARI with fever or cough were recruited at outpatient clinics in Marshfield, Wisconsin; southeastern Michigan (Ann Arbor and Detroit); Temple-Belton, Texas; Seattle, Washington; and Pittsburgh, Pennsylvania. Patients meeting the symptom criteria were eligible if duration of illness was ≤7 days and they had not received antiviral medication prior to enrollment. Recruitment and sample collection were performed by study personnel at each site and not influenced by the diagnosis of the treating physician. Consenting patients or their parents/guardians completed an enrollment interview to ascertain patient demographic characteristics, symptoms (fever, cough, fatigue, sore throat, nasal congestion, shortness of breath, wheezing), onset date, and subjective assessments of general health, current health status and self-reported influenza vaccination status.

Nasal and throat swabs (nasal only for children age <2 years) were collected and combined for influenza testing at network laboratories. This technique was selected because it is easier to collect and less uncomfortable for the patient and has been found to be as effective as nasopharyngeal swabs [17]. Presence of influenza was tested using real-time reverse transcription polymerase chain reaction (PCR) as previously described [16]. The parent study used a test-negative case-control design [18–20].

### Selection of study sample

Individuals enrolled in 2011–2012 from all 5 sites during periods of influenza circulation at each site were included in the analyses. That is, influenza circulation at each site was determined to be the time between date on which the first influenza positive case was enrolled and the last influenza-positive case was enrolled. Although participants may have reported onset of symptoms before or after this period; they were excluded from analysis. The total sample for all sites was 5,147. Some individuals were enrolled multiple times; all of those visits except the first enrollment were excluded (*N* = 71) reducing the sample to 5,076. Because symptoms of influenza vary between young children and older individuals, the primary analysis sample was restricted to enrollees ≥5 years of age, resulting in a final sample size of 4,173. Secondary analyses included children <5 years of age.

### CART analysis

Classification and Regression Trees (CART) [15] software was used to develop models that can classify subjects into various risk categories. Recursive partitioning, a non-parametric statistical method for multivariable data, uses a series of dichotomous splits, e.g., presence or absence of symptoms and other demographic variables, to create a decision tree, with the goal of correctly classifying members of the population, in this case, laboratory-confirmed influenza cases. Each independent variable is examined and a split is made to maximize the sensitivity and specificity of the classification, resulting in a decision tree. The objective of pruning is to develop a tree with the best size and lowest misclassification rate [15].

The CART method is able determine the complex interactions among variables in the final tree, in contrast to identifying and defining the interactions in a multivariable logistic regression model.

To begin the CART analysis, simple random sampling without replacement was used to split the sample into equal sized (50 %–50 %) developmental and validation samples. CART was applied first on a developmental sample then on a validation sample to assess the model’s generalizability and to evaluate the over fitting of the model to the developmental sample.

Several sets of candidate predictors were used to build the classification trees. Using several iterations, CART models were used to determine a clinically logical fit, based on sensitivity and specificity; the variables included those that were potentially related to risk of influenza such as, symptoms, self-reported vaccination status, personal and demographic variables and presence of chronic disease. The primary developmental and validation models were constructed for all participants ≥5 years, using self-reported vaccination status, household smoking status, and symptoms reported at enrollment: cough, fever, fatigue, wheezing, sore throat, nasal congestion, shortness of breath. The variables smoking status, age and presence of other high risk conditions were not included in this model.

The Gini Index method was used to split off the largest category into a separate group, with the default split size set to enable growing the tree. When the final tree was built, the tree was pruned, deleting the variables that did not further classify subjects, based on the variable importance score and the sensitivity, into an influenza group or no influenza group. Once a clinically meaningful structure on the CART evolved, pruning was discontinued. Hosmer-Lemeshow goodness of fit test confirmed the suitability of the trees.

Secondary analyses were constructed that included: 1) children 6–59 months of age, presenting within 2 days of onset of symptoms and included PCR-confirmed influenza status, self-reported vaccination status, household smoking status, and symptoms reported at enrollment: cough, fever, fatigue, wheezing, sore throat, nasal congestion, shortness of breath; and 2) adults ≥65 years old and individuals 5–64 years old with a high risk condition, presenting within 2 days of onset of symptoms and included PCR-confirmed influenza status, self-reported vaccination status, household smoking status, symptoms reported at enrollment: cough, fever, fatigue, wheezing, sore throat, nasal congestion, shortness of breath, and asthma diagnosis.

Receiver Operating Characteristics (ROC) curves and the area under the curve (AUC), sensitivity, specificity, positive and negative predictive values which were estimated using CART software were used to assess the performance of the CART model for the developmental and validation samples. The sensitivity from the CART model was determined using the final influenza positive terminal node and specificity was determined using the previous influenza negative terminal nodes.

### Additional analyses

In addition to the CART analyses, descriptive statistics were calculated as percentages for discrete variables and as means and standard deviations for continuous variables. Chi-square statistics were used to compare the distribution of symptoms and other discrete measures and Student’s t-tests were used to compare the continuous measures (i.e., age) between those with and without laboratory-confirmed influenza.

To support the CART findings, sensitivity analyses were conducted using multivariable regression analyses with a full model method, using the same set of variables used in the CART analysis for both developmental and validation samples, and for the full sample with all individuals ≥5 years of age. Positive and negative predictive values were calculated using sensitivity and specificity values from the CART model across a hypothetical range of influenza prevalence values (1–40 %) to reflect influenza seasons of varying severity (Table 3). The sensitivity and specificity, calculated using the predicted probability from the multivariable logistic regression for both developmental, validation and the full sample with the true classification of influenza, were obtained and are presented.

Statistical significance was defined as a two-sided *p* value <0.05. Data were analyzed using SAS v9.2 (SAS Institute, Inc., Cary, NC) and CART for the decision trees (Predictive Modeler) Software version 7.0.0.470 (Salford Systems, San Diego, CA).

## Results

Distributions of demographic variables for all enrollees with PCR-confirmed influenza or no influenza are shown in Table 1 and for the final developmental and validation samples are shown in Appendix 1: Table 4. All variables used in the tested models are shown. Individuals with PCR-confirmed influenza were more likely to report fever, cough and fatigue at enrollment and less likely to report household smoking, asthma diagnosis and were younger than those without influenza. In this cohort, antiviral use was low. Only 185 of 4173 enrollees (4.4 %) were prescribed an antiviral medication (15 % of cases vs. 6 % of non-cases were prescribed antivirals; Chi square *P* < 0.001). The use of antiviral medication among those ≥5 years old with a positive PCR test was 22 % (*n* = 40).

**Table 1:** Sociodemographic characteristics and symptoms of enrollees ≥ 5 years of age reported at enrollment, by Polymerase Chain Reaction (PCR)-confirmed Influenza status

	Overall				Developmental sample	Validation sample
Characteristics		PCR-negative for influenza	PCR-confirmed influenza	*P* value^a^		
	Total	*n* = 3531	*n* = 642		Total	Total
	*N* = 4173	(84.6 %)	(15.4 %)		(*N* = 2087)	(*N* = 2086)
	*N* (%)	*n* (%)	*n* (%)		*N* (%)	*N* (%)
Vaccinated by self-report	354 (8.7)	311 (87.8)	43 (12.2)	0.08	173 (8.5)	181 (8.9)
Unvaccinated	3714 (91.3)	3131 (84.3)	583 (15.7)			
Smoker^b^	436 (15.6)	386 (88.5)	50 (11.5)	0.07	215 (15.2)	221 (16)
Non-smoker	2358 (84.4)	2010 (85.2)	348 (14.8)			
Smoker in the household	481 (11.6)	428 (89.0)	53 (11.0)	0.004	223 (10.7)	258 (12.4)
No smoker in the household	3667 (88.4)	3081 (84.0)	586 (16.0)			
Asthma diagnosis	1098 (26.8)	949 (86.4)	149 (13.6)	0.04	543 (26.4)	555 (27.1)
No asthma diagnosis	3007 (73.3)	2521 (83.8)	486 (16.2)			
Any high risk condition	1147 (85.0)	1004 (87.5)	143 (12.5)	0.14	569 (84.4)	578 (85.5)
No high risk condition	203 (15.0)	170 (83.7)	33 (16.3)			
Symptoms reported at enrollment						
Fever	2910 (69.7)	2327 (80.0)	583 (20.0)	<.001	1461 (70)	1449 (69.5)
No fever	1263 (30.3)	1204 (95.3)	59 (4.7)			
Cough	3818 (91.5)	3189 (83.5)	629 (16.5)	<.001	1912 (91.6)	1906 (91.4)
No cough	355 (8.5)	342 (96.3)	13 (3.7)			
Fatigue	3610 (86.5)	3003 (83.2)	607 (16.8)	<.001	1795 (86)	1815 (87)
No fatigue	563 (13.5)	528 (93.8)	35 (6.2)			
Wheezing	1550 (37.1)	1292 (83.3)	258 (16.7)	0.08	773 (37)	777 (37.2)
No wheezing	2623 (62.9)	2239 (85.4)	384 (14.6)			
Sore throat	3105 (74.4)	2639 (85.0)	466 (15.0)	0.25	1543 (73.9)	1562 (74.9)
No sore throat	1068 (25.6)	892 (83.5)	176 (16.5)			
Nasal Congestion	3351 (80.3)	2823 (84.2)	528 (15.8)	0.18	1690 (81)	1661 (79.6)
No nasal congestion	821 (19.7)	707 (86.1)	114 (13.9)			
Shortness of breath	1899 (45.5)	1598 (84.2)	301 (15.8)	0.45	911 (43.7)	988 (47.4)
No shortness of breath	2272 (54.5)	1931 (85.0)	341 (15.0)			
Age, years, Mean (SD)	34.1 (22.2)	34.5 (22.2)	31.9 (21.8)	0.007	34.8 (22.3)	33.5 (22)

^a^For difference between influenza cases and controls

^b^Asked of those ≥18 years only

### Primary CART analyses

**Fig. 1:**
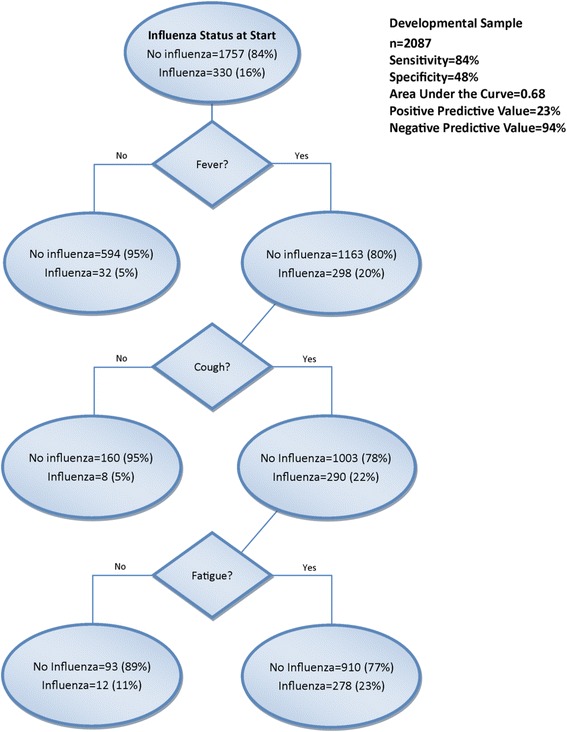
CART decision tree for the developmental sample for all enrollees ≥5 years for the outcome RT-PCR-confirmed Influenza

**Fig. 2:**
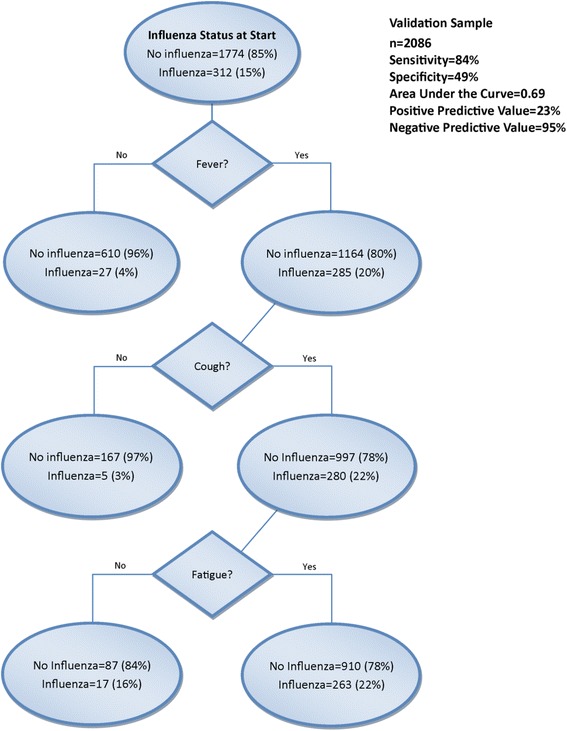
CART decision tree for the validation sample for all enrollees ≥5 years for the outcome RT-PCR-confirmed influenza

**Fig. 3:**
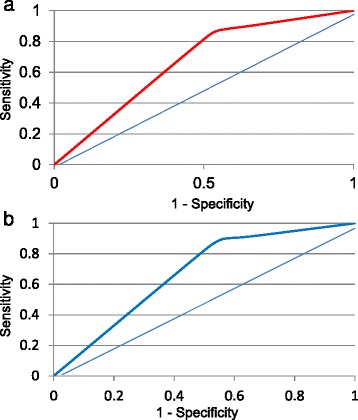
Receiver operating curve for CART algorithm on developmental sample for the outcome RT-PCR-confirmed Influenza. **a** Sensitivity = 278/330 = 84.2 %; Specificity = sum of all non-influenza subjects with negative symptoms in the terminal nodes/total non-influenza subjects, i.e. (93 + 160 + 594)/1757 = 48.2 %; Area under the Curve = 0.68. **b** Sensitivity = 84 %; Specificity = 49 %; Area under the Curve = 0.69

Figures 1 and 2 show the CART decision trees for the developmental and validation samples, respectively showing the conditions that would need to be present to predict influenza with maximum certainty for this sample. For the developmental sample, the sensitivity was 84 % and the specificity was 48 %. Positive predictive value (PPV) was 23 % and negative predictive value (NPV) was 94 % (Fig. 1). For the validation sample that examined the other half of the sample, the sensitivity was 84 % and the specificity was 49 % with a PPV of 23 % and NPV of 95 % (Fig. 2). The receiver operating characteristic (ROC) curves for the developmental and validation decision trees are shown in Fig. 3a, b, respectively, with area under the curve (AUC) =0.68 for the developmental sample and AUC = 0.69 for the validation sample. The misclassification rates for developmental and validation CART models were 16 % and 15 %, respectively. When the subjects were restricted to those who were enrolled within 2 days of illness onset, the model included fever and cough with a sensitivity of 89 % and a specificity of 50 %.

### Antiviral candidate analyses

**Fig. 4:**
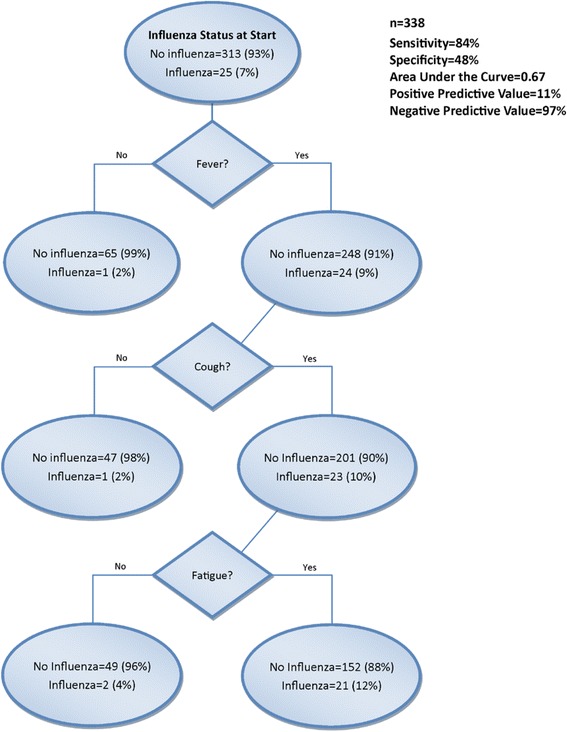
CART decision tree for those < 5 years old and presenting ≤2 days after onset of symptoms

**Fig. 5:**
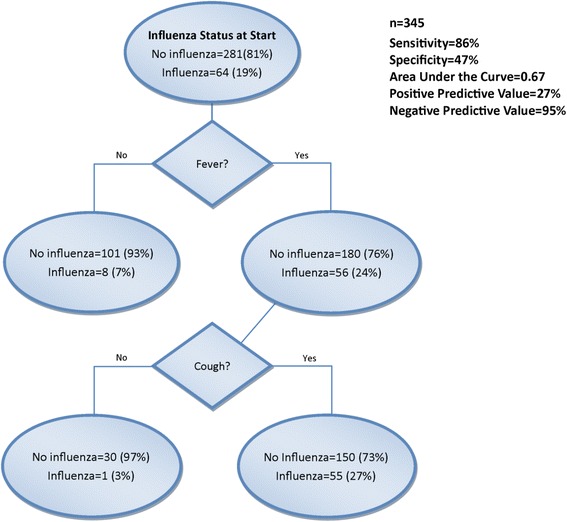
CART decision tree for those 65 years and older and those 5-64 years with a high risk condition presenting ≤2 days after onset of symptoms

For the model including only children <5 years old, the pruned CART decision tree (Appendix 2: Figure 4) contained fever, cough and fatigue with a sensitivity of 84 %, specificity of 48 %, PPV of 11 %, NPV of 97 % and an AUC =0.69. For the high risk model including individuals 5–64 years with a high risk condition and those ≥65 years, the CART decision tree (Appendix 3: Figure 5) contained fever and cough with a sensitivity of 86 %, specificity of 47 %, PPV of 27 %, NPV of 95 % and an AUC = 0.67. The average log-likelihoods to test the goodness of fit are shown in Appendix 4: Table 5.

### Comparison of CART with multivariable logistic regression

For comparison of CART with traditional multivariable logistic regression analyses, Table 2 shows the odds ratios (ORs) and 95 % confidence intervals (CIs); fever, cough and fatigue significantly increased the likelihood of PCR-confirmed influenza while exposure to household smoking decreased the likelihood of influenza. Shortness of breath, wheezing, sore throat and nasal congestion were not related to influenza. For the full nine-variable logistic regression equation for the observed prevalence of influenza of 15.4 %, the resultant values from the predicted probability were 82 % sensitivity, 52 % specificity, 24 % PPV, 94 % NPV and c-statistic (AUC) = 0.70. The multivariable stepwise logistic regression model resulted in the same significance of those four variables from the full model. The c-statistic = 0.69 for the step-wise selection model.

**Table 2:** Likelihood of Polymerase Chain Reaction (PCR)-confirmed Influenza by logistic regression among 4,173 enrollees ≥5 years of age

Variable	Logit Model	
	Odds Ratio	95 % CI
Vaccinated by self-report	0.73	0.52–1.03
Smoker in the household	0.62	0.45–0.85
Symptoms reported at enrollment		
Fever	5.33	4.01–7.10
Cough	7.32	4.14–12.9
Fatigue	2.32	1.58–3.41
Wheezing	0.99	0.81–1.21
Sore throat	0.84	0.69–1.03
Nasal congestion	1.06	0.84–1.35
Shortness of breath	0.86	0.71–1.05

### Impact of varying prevalence

The prevalence of influenza during 2011–2012 influenza season among enrollees ranged from <3 % in January to a peak of 23 % in March, indicating a late and relatively light season. Therefore, we compared PPV across a range of influenza prevalence values to determine the value of the decision aid in milder or more severe seasons. Using consistent values of 84 % sensitivity and 48 % specificity, PPV ranged from 1.6 to 51.9 % and NPV ranged from 99.7 to 81.8 % when prevalence was varied from 1 to 40 % (Table 3).

**Table 3:** Positive Predictive Values (PPV) and Negative Predictive Values (NPV) across a range of influenza prevalence using the Sensitivity (84 %) and Specificity (48 %) of the diagnostic model based on CART analyses

	Prevalence^a^	PPV, %	NPV, %
Hypothetical ranges of influenza prevalence	1.0	1.61	99.7
	10.0	15.2	96.4
	15.0	22.2	94.4
	20.0	28.8	92.3
	25.0	35.0	90.0
	30.0	40.9	87.5
	35.0	46.5	84.8
	40.0	51.9	81.8
2011–2012 influenza prevalence in US Influenza Vaccine Effectiveness Network	15.4	22.7	94.3

Note: *CART* classification and regression trees

^a^Per one hundred

## Discussion

Eighty-five percent of influenza positive cases in this study were not prescribed an antiviral medication; whereas, 94 % of influenza negative cases were not prescribed an antiviral medication. While empiric treatment of certain patients is recommended, influenza prediction tools may be a useful adjunctive approach to improving appropriate use of antiviral medication.

A range of techniques, including clinical judgment [21], clinical decision rules [13, 22, 23], and CART [14], has been used to predict the likelihood of seasonal influenza among individuals presenting for outpatient treatment of an ARI. In multivariable regression analyses, Monto et al. found that presence of fever and cough in adults and adolescents in ambulatory settings best predicted influenza confirmed by cultures, 4-fold increase in antibody titer, immunofluorescence, or PCR [21]. These findings were confirmed among children ≥5 years of age [23]. Subsequent work by Stein et al. [22], also using multivariable regression analyses and PCR to confirm influenza, found no improvement over clinical judgment of either rapid testing or a clinical prediction rule in which specific symptoms were assigned scores.

The advantages of CART are that recursive partitioning does not make any distributional assumptions about the modeled variables, and that it accounts for multi-level interactions among variables. We found a similar area under the curve with CART and logistic regression, but CART used fewer variables.

Afonso et al. used CART to develop a model predicting low and high risk of influenza among 459 patients combining data from two studies from different countries [14]. Their model included temperature >37.3 °C and presence of chills or sweating and produced an AUC of .75-.76. In comparison, we report a decision tree using four symptoms (fever, cough, fatigue and shortness of breath) and presence of household smoke with an AUC of .68–.69. We did not ask about chills/sweating. Our model had a PPV of 23 % and NPV of 95 %. Testing the sensitivity of 81 % and specificity of 52 % of the CART model across a range of prevalence values indicated that, in a more typical influenza season with a hypothetical prevalence of 25 %, the PPV increased to 36 % with a 91 % NPV.

In a review of clinical decision rules for diagnosis of influenza, Ebell and Afonso [24] presented suggestions for future studies of this topic, of which the present study addresses several. For example, the authors suggested using PCR as the reference standard for the detection of influenza, an adequate sample size, and a broad range of patients. PCR was used exclusively for confirmation of influenza in this study of 4,173 patients ≥5 years of age from across the U.S., thus addressing some of these conditions. The value of this analysis is the ability to identify, from among a group of patients with ARI and onset of fever or cough within 7 days, those patients with fever, cough, fatigue, as having influenza with 84 % sensitivity. Thus, the algorithm is good at identifying a group who do not need testing or antivirals (i.e., algorithm negative); however, among those “positive” by the algorithm, laboratory testing or treatment based on clinical judgement is indicated. Our additional analyses that limited the population to those whose onset of symptoms was 2 or fewer days before presentation, with a high risk condition or high risk age group, could be used to develop guidance for prescribing antivirals based on symptoms alone. CART analyses support previous studies using multivariable regression to predict influenza from symptoms [21–23], but CART offers the advantage of requiring fewer variables for input into a clinical decision tool.

Determining a low-risk group in whom neither testing nor antivirals is warranted could save resources and avoid inappropriate antiviral prescribing and concomitant concerns about the development of viral resistance. A threshold approach to clinical decision-making was advocated by Pauker and Kassirer [25], with one threshold for testing and another for treating. For influenza, these testing and treatment thresholds vary by country and are lower for US compared to Swiss physicians [26]. We consider the 95 % NPV sufficiently good to rule-out influenza and avoid testing in this group. In light of the findings that use of antiviral medication among patients with influenza is low [27], and the cost of PCR testing is high [28, 29], a clinical decision aid based on symptoms offers the opportunity to promote appropriate testing during the times when it is most cost-effective, and increase antiviral medication use among those most likely to benefit, while avoiding a large increase in use in those who will not benefit.

### Limitations and strengths

The limitations of this study are that the data are from a single year, in which influenza circulated later than usual and was less prevalent than normal. Moreover, influenza vaccine uptake and effectiveness may affect the model’s predictive validity. CART does not provide a p-value to test significance. Conversely, an advantage of CART is its ability to examine complex higher order interactions among variables. The analysis is strengthened by the inclusion of data from a large sample of outpatients from five sites spanning the U.S. However, it did not include developing countries or tropical regions with differing etiologies of infectious diseases.

## Conclusions

Although CDC recommends empiric use of antivirals, their use remains low. Recursive partitioning using CART analyses to establish a clinical decision algorithm for influenza has good sensitivity and NPV, but limited PPV. Thus, it is good at identifying a group who do not need testing or antivirals; however, among those “positive” by the algorithm, laboratory testing or treatment based on clinical judgment is indicated. Further testing during additional influenza seasons may help to determine how this algorithm could be used to optimize decisions on laboratory testing and antiviral use in patients with ARI.

## Appendix 1

**Table 4:** Sociodemographic characteristics and symptoms of enrollees ≥ 5 years of age reported at enrollment for developmental and validation samples

	Developmental sample Total (*N* = 2087)	Validation sample Total (*N* = 2086)
Characteristics	*N* (%)	*N* (%)
Vaccinated by self-report	173 (8.5)	181 (8.9)
Smoker	215 (15.2)	221 (16)
Smoker in the household	223 (10.7)	258 (12.4)
Asthma diagnosis	543 (26.4)	555 (27.1)
Any high risk condition	569 (84.4)	578 (85.5)
Symptoms reported at enrollment		
Fever	1461 (70)	1449 (69.5)
Cough	1912 (91.6)	1906 (91.4)
Fatigue	1795 (86)	1815 (87)
Wheezing	773 (37)	777 (37.2)
Sore throat	1543 (73.9)	1562 (74.9)
Nasal Congestion	1690 (81)	1661 (79.6)
Shortness of breath	911 (43.7)	988 (47.4)
Age, years, Mean (SD)	34.8 (22.3)	33.5 (22)

## Appendix 2

**Table 5:** Tests of goodness of fit of each CART model using log-likelihood

Tree with risk factor	Average log likelihood	Sensitivity	Specificity	Hosmer-Lemeshow Statistic *p* value
Fever	−0.421	90	34	1
Fever, Cough	−0.406	88	43	1
Fever, Cough, Fatigue	−0.403	84	48	1
Fever, Cough, Fatigue, Household smoke, smoking, shortness of breath	−0.401	81	52	0.99

## Abbreviations

ARI: Acute respiratory infections
AUC: Area under the curve
CART: Classification and regression trees
CI: Confidence intervals
NPV: Negative predictive value
OR: Odds ratio
PCR: Polymerase chain reaction
PPV: Positive predictive value
ROC: Receiver operating characteristics
US Flu VE Network: U.S. Influenza Vaccine Effectiveness Network

## References

[1] Thompson WW, Shay DK, Weintraub E, Brammer L, Bridges CB, Cox NJ, Fukuda K (2004). Influenza-associated hospitalizations in the United States. JAMA.

[2] Molinari NA, Ortega-Sanchez IR, Messonnier ML, Thompson WW, Wortley PM, Weintraub E, Bridges CB (2007). The annual impact of seasonal influenza in the US: measuring disease burden and costs. Vaccine.

[3] Heneghan CJ, Onakpoya I, Jones MA, Doshi P, Del Mar CB, Hama R, Thompson MJ, Spencer EA, Mahtani KR, Nunan D (2016). Neuraminidase inhibitors for influenza: a systematic review and meta-analysis of regulatory and mortality data. Health Technol Assess.

[4] Jefferson T, Jones MA, Doshi P, Del Mar CB, Hama R, Thompson MJ, Spencer EA, Onakpoya I, Mahtani KR, Nunan D (2014). Neuraminidase inhibitors for preventing and treating influenza in healthy adults and children. Cochrane Database Syst Rev.

[5] Fiore AE, Fry A, Shay D, Gubareva L, Bresee JS, Uyeki TM. Antiviral agents for the treatment and chemoprophylaxis of influenza: recommendations of the Advisory Committee on Immunization Practices (ACIP). Recommendations and Reports. MMWR. 2011;60(RR01);1–24. 21248682

[6] Dobson J, Whitley RJ, Pocock S, Monto AS (2015). Oseltamivir treatment for influenza in adults: a meta-analysis of randomised controlled trials. Lancet.

[7] Hernan MA, Lipsitch M (2011). Oseltamivir and risk of lower respiratory tract complications in patients with flu symptoms: a meta-analysis of eleven randomized clinical trials. Clin Infect Dis.

[8] Hsu J, Santesso N, Mustafa R, Brozek J, Chen YL, Hopkins JP, Cheung A, Hovhannisyan G, Ivanova L, Flottorp SA (2012). Antivirals for treatment of influenza: a systematic review and meta-analysis of observational studies. Ann Intern Med.

[9] Havers F, Thaker S, Clippard JR, Jackson M, McLean HQ, Gaglani M, Monto AS, Zimmerman RK, Jackson L, Petrie JG (2015). Use of Influenza Antiviral Agents by Ambulatory Care Clinicians During the 2012–2013 Influenza Season. Clin Infect Dis.

[10] Rapid Diagnostic Testing for Influenza: Information for Clinical Laboratory Directors http://www.cdc.gov/flu/professionals/diagnosis/rapidlab.htm. Accessed 6 June 2016.

[11] Bright T, Wong A, Dhurjati R, Bristow E, Bastian L, Coeytaux R, Samsa G, Hasselblad V, Williams J, Musty M (2012). Effect of clinical decision-support systems: a systematic review. Ann Intern Med.

[12] Patel RB, Mathur MB, Gould M, Uyeki TM, Bhattacharya J, Xiao Y, Khazeni N (2014). Demographic and clinical predictors of mortality from highly pathogenic avian influenza A (H5N1) virus infection: CART analysis of international cases. PLoS One.

[13] Ebell MH, Afonso AM, Gonzales R, Stein J, Genton B, Senn N (2012). Development and validation of a clinical decision rule for the diagnosis of influenza. J Am Board Fam Med.

[14] Afonso AM, Ebell MH, Gonzales R, Stein J, Genton B, Senn N (2012). The use of classification and regression trees to predict the likelihood of seasonal influenza. Fam Pract.

[15] Breiman L, Friedman JH, Olshen RA, Stone CJ (1984). Classification and regression trees (Wadsworth statistics/probability).

[16] Ohmit SE, Thompson MG, Petrie JG, Thaker SN, Jackson ML, Belongia EA, Zimmerman RK, Gaglani M, Lamerato L, Spencer SM (2014). Influenza vaccine effectiveness in the 2011–2012 season: protection against each circulating virus and the effect of prior vaccination on estimates. Clin Infect Dis.

[17] Spencer S, Gaglani M, Naleway A, Reynolds S, Ball S, Bozeman S, Henkle E, Meece J, Vandermause M, Clipper L (2013). Consistency of influenza A virus detection test results across respiratory specimen collection methods using real-time reverse transcription-PCR. J Clin Microbiol.

[18] Jackson ML, Nelson JC (2013). The test-negative design for estimating influenza vaccine effectiveness. Vaccine.

[19] Foppa IM, Haber M, Ferdinands JM, Shay DK (2013). The case test-negative design for studies of the effectiveness of influenza vaccine. Vaccine.

[20] De Serres G, Skowronski DM, Wu XW, Ambrose CS. The test-negative design: validity, accuracy and precision of vaccine efficacy estimates compared to the gold standard of randomised placebo-controlled clinical trials. Euro Surveillance 2013; 18(37). 10.2807/1560-7917.es2013.18.37.20585 24079398

[21] Monto AS, Gravenstein S, Elliott M, Colopy M, Schweinle J (2000). Clinical signs and symptoms predicting influenza infection. Arch Intern Med.

[22] Stein J, Louie J, Flanders S, Maselli J, Hacker JK, Drew WL, Gonzales R (2005). Performance characteristics of clinical diagnosis, a clinical decision rule, and a rapid influenza test in the detection of influenza infection in a community sample of adults. Ann Emerg Med.

[23] Ohmit SE, Monto AS (2006). Symptomatic predictors of influenza virus positivity in children during the influenza season. Clin Infect Dis.

[24] Ebell MH, Afonso A (2011). A systematic review of clinical decision rules for the diagnosis of influenza. Ann Fam Med.

[25] Pauker SG, Kassirer JP (1980). The threshold approach to clinical decision making. N Engl J Med.

[26] Ebell MH, Locatelli I, Senn N. A novel approach to the determination of clinical decision thresholds. Evid Based Med. 2015. 10.1136/ebmed-2014-110140 25736042

[27] Havers FP, Flannery B, Clippard JR, Gaglani M, Zimmerman RK, Jackson LA, Petrie JG, McLean HQ, Nowalk MP, Jackson ML, et al. Use of influenza antiviral medications among outpatients at high risk for influenza-associated complications during the 2013–14 influenza season. Clin Infect Dis. 2015. 10.1093/cid/civ146 PMC4542604 25722198

[28] Nelson RE, Stockmann C, Hersh AL, Pavia AT, Korgenksi K, Daly JA, Couturier MR, Ampofo K, Thorell EA, Doby EH (2015). Economic analysis of rapid and sensitive polymerase chain reaction testing in the emergency department for influenza infections in children. Pediatr Infect Dis J.

[29] Nicholson KG, Abrams KR, Batham S, Medina MJ, Warren FC, Barer M, Bermingham A, Clark TW, Latimer N, Fraser M (2014). Randomised controlled trial and health economic evaluation of the impact of diagnostic testing for influenza, respiratory syncytial virus and Streptococcus pneumoniae infection on the management of acute admissions in the elderly and high-risk 18- to 64-year-olds. Health Technol Assess.

